# Allele-specific polymerase chain reaction can determine the diplotype of *NUDT15* variants in patients with childhood acute lymphoblastic Leukemia

**DOI:** 10.1038/s41598-023-27720-2

**Published:** 2023-01-10

**Authors:** Chih-Hsiang Yu, Ya-Hsuan Chang, Der-Shiun Wang, Shiann-Tarng Jou, Chien-Yu Lin, Kai-Hsin Lin, Meng-Yao Lu, Kang-Hsi Wu, Chao-Neng Cheng, Hsiu-Hao Chang, Shu-Wei Chou, Min-Yu Su, Yu-Ling Ni, Pei-Yuan Xu, Dong-Tsamn Lin, Shu-Wha Lin, Hsuan-Yu Chen, Yung-Li Yang

**Affiliations:** 1 Institute of Statistical Science Academia Sinica, Taipei, Taiwan; 2 Department of Pediatrics, Tri-Service General Hospital, National Defense Medical Center, Taipei, Taiwan; 3 Graduate Institute of Clinical Medicine, College of Medicine, National Taiwan University, Taipei, Taiwan; 4 Department of Pediatrics, National Taiwan University Children’s Hospital, Taipei, Taiwan; 5 Department of Pediatrics, College of Medicine, National Taiwan University, Taipei, Taiwan; 6 Department of Pediatrics, Chung Shan Medical University Hospital, Taichung, Taiwan; 7 School of Medicine, Chung Shan Medical University, Taichung, Taiwan; 8 Department of Pediatrics, National Cheng Kung University Hospital, Tainan, Taiwan; 9 Department of Pediatrics, College of Medicine, National Cheng Kung University, Tainan, Taiwan; 10 Department of Pediatrics, China Medical University Children’s Hospital, Taichung, Taiwan; 11 Department of Laboratory Medicine, National Taiwan University Hospital, Taipei, Taiwan; 12 Departments of Clinical Laboratory Sciences and Medical Biotechnology, National Taiwan University, Taipei, Taiwan; 13 Department of Laboratory Medicine, College of Medicine, National Taiwan University, Taipei, Taiwan

**Keywords:** Haematological cancer, Haplotypes

## Abstract

Mercaptopurine intolerance is an adverse effect of mercaptopurine administration in pediatric patients with acute lymphoblastic leukemia (ALL). *NUDT15* variants have emerged as major determinants of mercaptopurine intolerance, especially in the Asian population. Two variants, c.55_56insGAGTCG in exon 1 and c.415C > T in exon 3, were commonly detected in the same allele, named NUDT15*1/*2. Although rare, compound heterozygous mutations also occur, with the two variants on different alleles (NUDT15*3/*6), which may confer tolerance to considerably lesser mercaptopurine dosage. Sanger sequencing or pyrosequencing can determine the *NUDT15* variants but not the phase. Here, we designed an allele-specific PCR (AS-PCR) with locked nucleic acid-modified primers. A cohort of 63 patients harboring heterozygous c.55_56insGAGTCG and c.415C > T *NUDT15* variations was selected for haplotyping using AS-PCR. Of the 63 patients, 60 harbored the NUDT15*1/*2 variant and three harbored compound heterozygous mutations, including two *NUDT1*5*3/*6 and one NUDT15*2/*7 variants. These findings suggest that AS-PCR can determine *NUDT15* diplotype and identify patients with compound heterozygous *NUDT15* variants, which may enable precise genetic diagnosis of *NUDT15*. Nevertheless, a larger clinical trial is required to understand the clinical significance of NUDT15*3/*6 in pediatric patients with ALL because of its low incidence rate and challenges in detecting this variant.

## Introduction

Thiopurines, including azathioprine, 6-thioguanine (6-TG), and 6-mercaptopurine (6-MP), are effective anti-inflammatory, anticancer, and immunosuppressive drugs that are widely administered for different clinical indications^1^. In pediatric acute lymphoblastic leukemia (ALL), 6-MP is widely used in the induction, consolidation (with high-dose methotrexate), and maintenance phases^2–5^. It is a prodrug that is converted to the active form upon metabolization^6^. Therefore, the proteins involved in the metabolic pathways might prolong the half-life of 6-MP, thereby augmenting its side effects, especially neutropenia^7^. Polymorphisms in the thiopurine methyltransferase (*TPMT*) gene have been linked to susceptibility to thiopurine-induced marrow suppression in patients. Preemptive *TPMT* genotype-guided dosing is a successful example of genetics-based precision medicine for treating childhood ALL, which has been used in Western countries for more than 30 years^8–11^.

Asian pediatric patients with ALL experience more thiopurine-induced toxicity than Europeans, despite a lower frequency of *TPMT* mutations. A genome-wide association study reported a missense variant in *NUDT15* (*rs116855232*, referred to as c.415C > T or p.Arg139Cys) strongly associated with thiopurine-related myelosuppression in patients with inflammatory bowel disease in a Korean population^12^. In addition, *NUDT15* c.415C > T was demonstrated to be associated with mercaptopurine intolerance in childhood ALL by Yang et al.^13^. *NUDT15* encodes a nucleoside diphosphatase that dephosphorylates thioguanine nucleotides, thereby preventing their incorporation into DNA^14^. Moriyama et al. sequenced all three coding regions of *NUDT15* and identified more comprehensive genetic variants of this gene. Several *NUDT15* variants associated with decreased diphosphatase activity have been reported, and haplotypes with different combinations of the variants have been assigned star allele numbers proposed by Moriyama et al.^15^. The loss-of-function NUDT15*2 allele is characterized by a missense variant in exon 3 (NM_018283.4:c.415C > T, p.Arg139Cys, rs116855232; also present in NUDT15*3) and an in-frame insertion in exon 1 (NM_018283.4:c.55_56GAGTCG, p.V18_V19insGV, rs746071566; also present independently as NUDT15*6)^11,15^.

Patients with heterozygous c.415C > T and c.55_56GAGTCG are either heterozygous (NUDT15*1/*2) or compound heterozygous (NUDT15*3/*6) for this mutation^16^. Patients with NUDT15*3/*6 are possibly intermediate metabolizers^11^. In theory, patients with two defective alleles might be poor metabolizers; for example, one patient with NUDT15*3/*6 was reported to tolerate a very low dose of mercaptopurine^16^. However, the currently available common methods cannot define diplotypes in complex genetic alterations. Hence, a simple genetic diagnostic method is required to identify the patients with NUDT15*3/*6 in daily clinical practice. In our previous study, we elucidated the *NUDT15* diplotypes in patients with multiple heterozygous variants by performing next-generation sequencing (NGS) using *NUDT15* cDNA. However, targeted sequencing of *NUDT15* cDNA cannot be used in routine clinical practice because of the cost and long turn-around time (TAT)^16^. In contrast, allele-specific PCR (AS-PCR) is a conceptually simple, single nucleotide polymorphism (SNP)-based genotyping strategy based on the position of the 3′ base of a PCR primer to match one SNP allele and accurately extend only the correctly matched primer under stringent conditions. Locked nucleic acid (LNA) is an RNA nucleotide analog with a methylene bridge connecting the 2' oxygen and 4' carbon. This bridge restricts the ribose in the C3′-endo conformation, thereby enhancing the stability of LNA and increasing the hybridization melting temperature (Tm)^17^. LNA hybridizes with its complementary sequence, with high affinity over DNA or RNA oligonucleotides, which makes LNA oligos an excellent tool for distinguishing single nucleotides^18^.

In this study, AS-PCR was performed using LNA primers to determine the diplotype of *NUDT15* in pediatric ALL patients who harbor heterozygous c.415C > T and c.55_56GAGTCG mutations. Although most patients were NUDT15*1/*2, there were two patients with *NUDT1*5*3/*6 identified by AS-PCR.

## Methods

### Patients

In total, 449 patients with pediatric ALL (aged < 18 years) were enrolled in this study between April 1997 and December 2021. We performed Sanger sequencing to screen for *NUDT15* variants and selected 63 patients with heterozygous c.55_56insGAGTCG and c.415C > T mutations^16,19^. The study was performed in accordance with the guidelines of the Declaration of Helsinki and approved by the Institutional Review Board of the National Taiwan University Hospital (201510016RIND). Written informed consent was obtained from parents, guardians, or patients.

### Genomic DNA extraction

White blood cells were purified from whole blood samples using RBC lysis buffer according to the manufacturer’s instructions (Thermo Fisher Scientific, Piscataway, NJ, USA). Genomic DNA was extracted from white blood cells using a phenol/chloroform-based method as described previously^20^, and the concentration of the extracted DNA was measured using a NanoDrop 1000 spectrophotometer (Thermo Fisher Scientific).

### *NUDT15* genotyping

**Figure 1:**
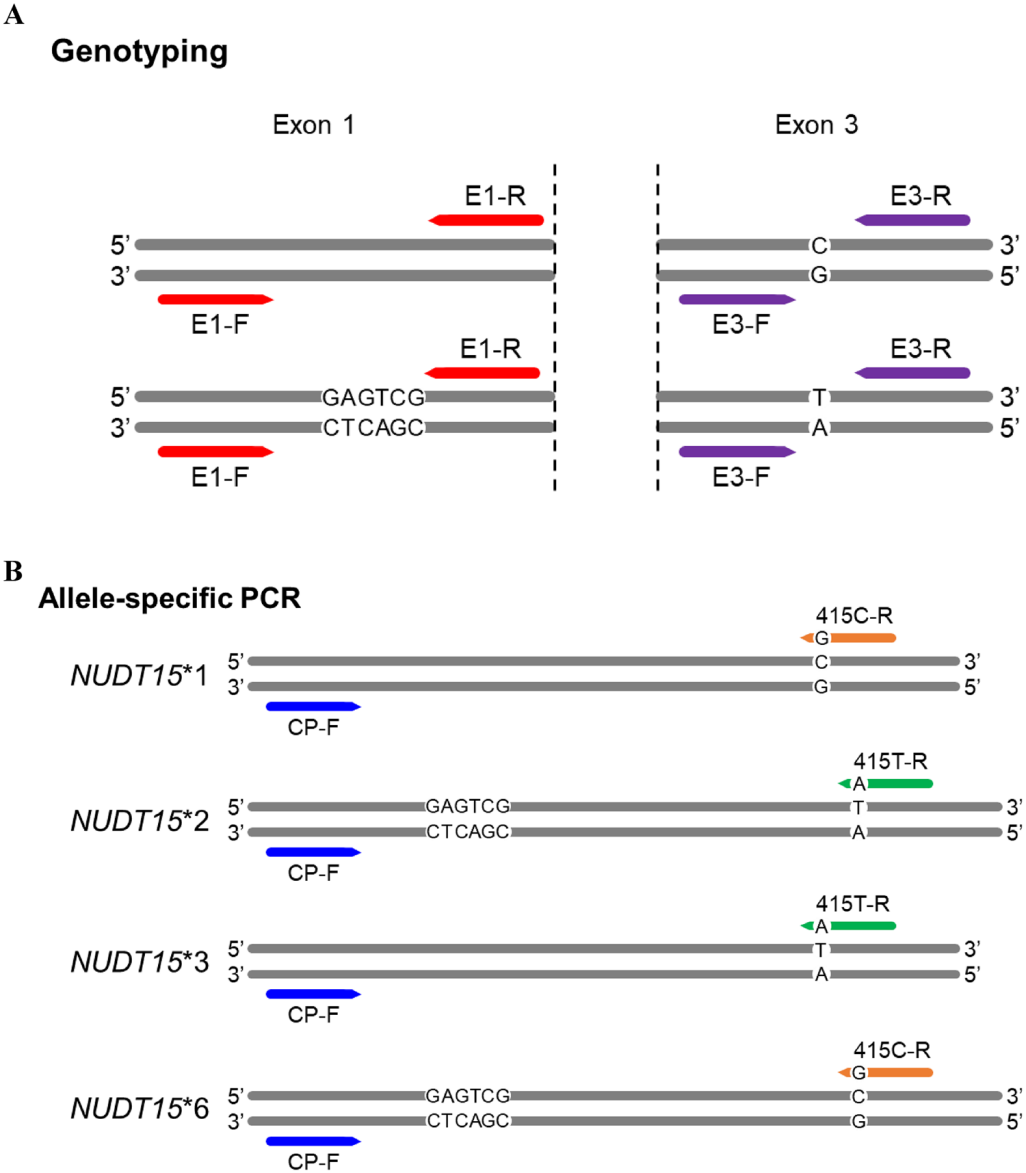
Primer designs for *NUDT15* genotyping and allele-specific PCR. (**A**) Two primer sets, E1-F/E1-R and E3-F/E3-R, were used to amplify exons 1 and 3 of *NUDT15,* respectively. (**B**) Two sets of locked nucleic acid (LNA) primers were used to amplify samples with heterozygous *NUDT15* c.55_56insGAGTCG and c.415C > T to differentiate between NUDT15*1/*2 and NUDT15*3/*6.

The method of *NUDT15* genotyping is published elsewhere^19^ and is shown in Fig. 1A. The patients harboring heterozygous c.55_56GAGTCG and c.415C > T were further analyzed using AS-PCR.

### AS-PCR analysis of *NUDT15*

The *NUDT15* c.415C and c.415T alleles were specifically amplified using AS-PCR. Each PCR reaction included 10 ng genomic DNA, 800 nM dNTP, 1 × PrimeSTAR GXL buffer, 200 nM forward (common primer, CP-F), 200 nM reverse primer (415C-R for c.415C allele or 415T-R for c.415T allele), and 0.75 U PrimeSTAR GXL DNA polymerase (Takara Bio, San Jose, CA, USA) (Fig. 1B). Thermocycling was performed under the following conditions: 98℃ for 5 min, 35 cycles of 98 ℃ for 10 s and 68 ℃ for 1.5 min, and final extension at 68℃ for 5 min. The PCR products were analyzed by performing gel electrophoresis on 1% agarose gel, and an 8.2 kb band was observed in case of successful PCR amplification. Gels were imaged by SmartView Pro Imager (Major Science, UVCI-1100). Images were acquired with 312 nm UV light at 3 to 5 s exposure times with the SmartView Pro 1100 Imager system (Major Science) and stored in a .JPEG format. The .JPEG files were directly used for presentation. PCR clean-up reaction was performed as follows prior to sequencing: 5 μL PCR product was mixed with 1 U FastAP (Thermo Fisher Scientific) and 10 U exonuclease I (Thermo Fisher Scientific) and incubated at 37℃ for 15 min, followed by 15 min incubation at 85℃. The purified PCR products were sequenced using the E1-S primer. The sequencing results were aligned to the NCBI GenBank entry NG_047021.1 using the SnapGene 4.1.3 software (GSL Biotech LLC, San Diego, CA, USA). The *NUDT15* diplotype was determined to be NUDT15*1/*2 when c.55_56insGAGTCG and c.415C > T were located on the same allele or NUDT15*3/*6 when c.55_56insGAGTCG and c.415C > T were located on the different alleles. Detailed information on the primers is listed in Table 1.

**Table 1:** The sequences of the primers used for *NUDT15* genotyping and allele-specific PCR. The underlined nucleotides indicate substitution by locked nucleic acid.

Primer sets	Primer name	Sequence (5′ to 3′)
*NUDT15* genotyping		
Exon 1	E1-F	CAAAGCACAACTGTAAGCGACT
	E1-R	GCAAAGACCTCGCCTGACCCA
Exon 3	E3-F	AGCCAAGCAAATGCAAAGCA
	E3-R	TGGCTGAAAGAGTGGGGGATA
Allele-specific PCR (AS PCR)		
415C AS-PCR	CP-F	CAAAGCACAACTGTAAGCGACT
	415C-R	AGCCTTGTTCTTTTAAACAACG
415T AS-PCR	CP-F	CAAAGCACAACTGTAAGCGACT
	415T-R	AGCCTTGTTCTTTTAAACAACA
Sequencing	E1-S	GGAAGGAAACACGGCATTC

## Results

### Sanger sequencing for screening *NUDT15* variants

**Figure 2:**
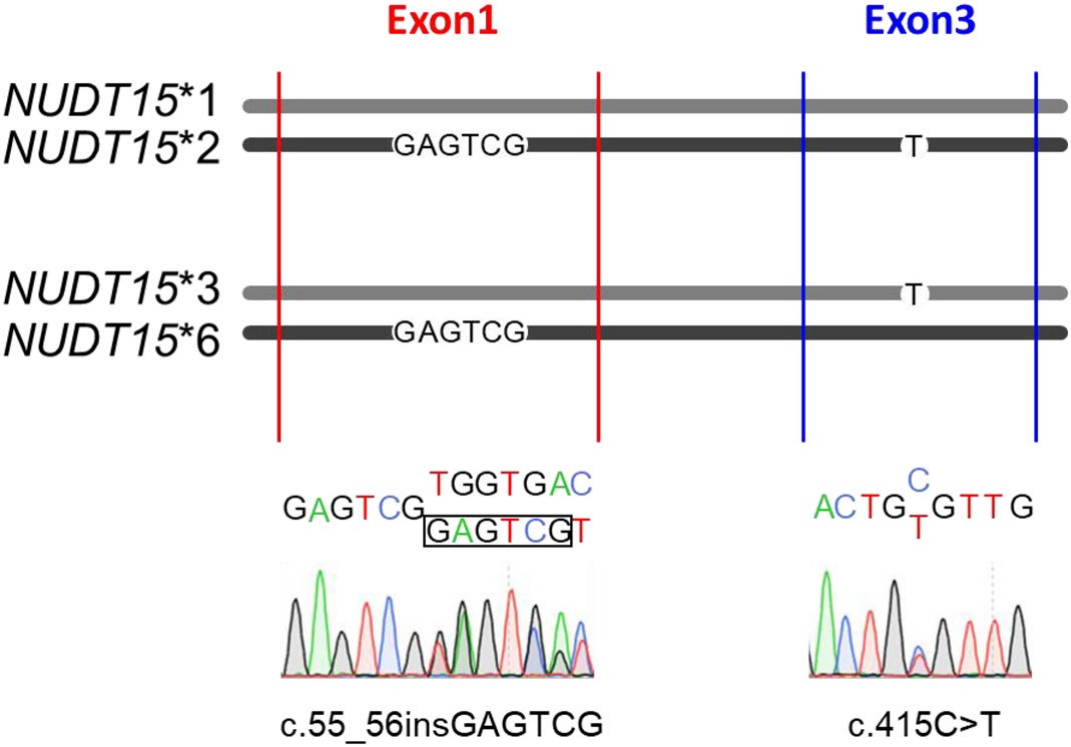
Results of Sanger sequencing of samples with both heterozygous c.55_56insGAGTCG and c.415C > T. The *NUDT15* exon 1 (red line) and exon 3 (blue line) regions are amplified separately, and the corresponding sequencing results are shown below. Both NUDT15*1/*2 and NUDT15*3/*6 showed identical Sanger sequencing signals.

Sanger sequencing to analyze heterozygous c.55_56insGAGTCG and c.415C > T variants (Fig. 2) identified a total of 63 patients harboring the heterozygous c.55_56insGAGTCG and c.415C > T variants (14%, 63/449). These patients included the patients (n = 37) evaluated in our previous publications^16,19^. The *NUDT15* diplotypes of these patients in the previous study were NUDT15*1/*2 (n = 35), NUDT15*3/*6 (n = 1, ID898) and NUDT15*2/*7 (n = 1, ID341)^16,19,21^. The genotype based on *NUDT15* expression of all 63 patients is shown in Table 2.

**Table 2:** Distribution of *NUDT15* diplotypes.

Diplotypes	Number (%)
Genotyping	
*1/*1	320 (71.3)
*1/*3	45 (10.0)
*1/*5	8 (1.8)
*1/*6	6 (1.3)
*1/*7	3 (0.7)
*2/*2	2 (0.4)
*2/*3	1 (0.2)
*2/*6	1 (0.2)
AS-PCR analysis	
*1/*2	60 (13.4)
*3/*6	2 (0.4)
*2/*7	1 (0.2)

### Distinguishing NUDT15*1/*2 from *3/*6 using AS-PCR

**Figure 3:**
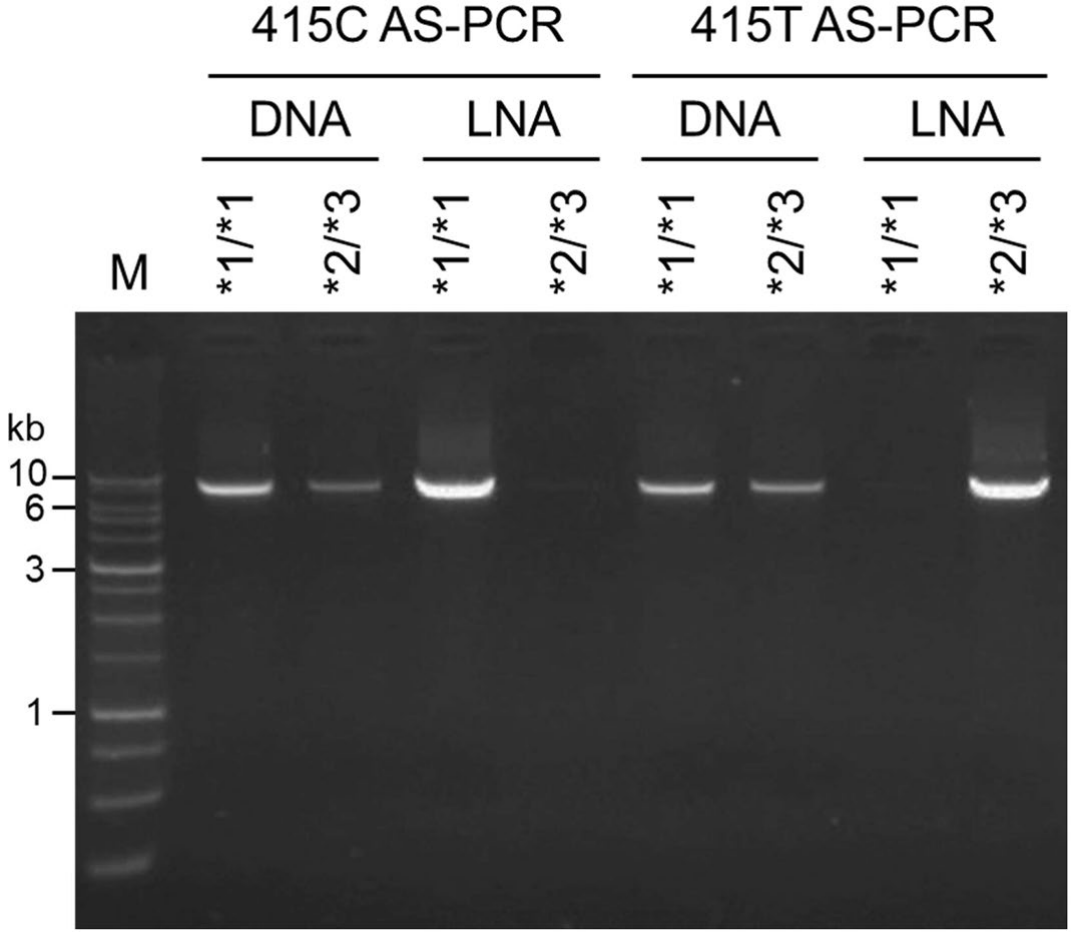
Comparison of allele-specific PCR (AS-PCR) using LNA or DNA primers. *NUDT15* was amplified using AS-PCR with LNA or DNA primers. Agarose gel electrophoresis of equal volumes of PCR products amplified from NUDT15*1/*1 (homozygous c.415C) and NUDT15*2/*3 (homozygous c.415T) samples; M, 1 kb DNA marker; kb, kilobase.

**Figure 4:**
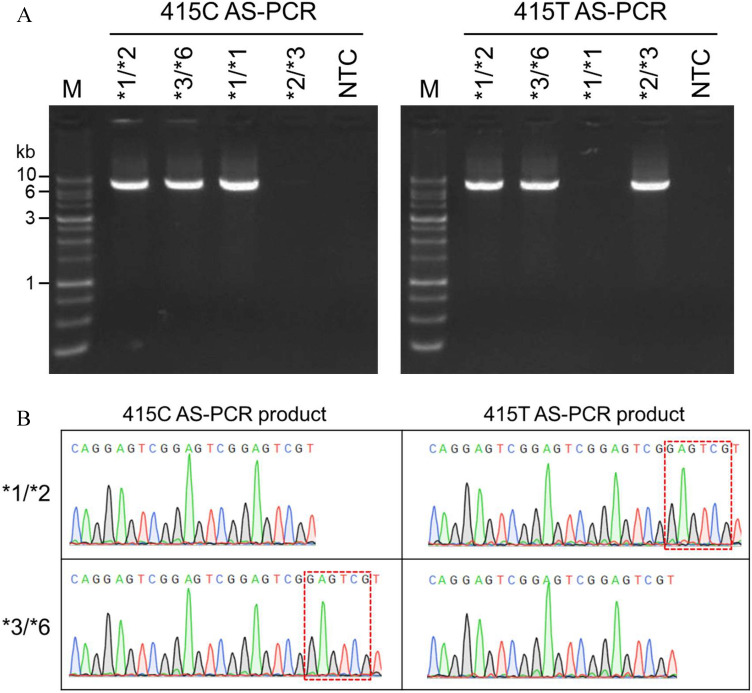
*NUDT15* diplotype analysis using allele-specific PCR (AS-PCR). (**A**) Samples with NUDT15*1/*2, *3/*6, *1/*1, and *2/*3 were amplified using 415C AS-PCR and 415T AS-PCR, and the PCR products were analyzed using 1% agarose gel electrophoresis. (**B**) Results of Sanger sequencing of AS-PCR product amplified from NUDT15*1/*2 and *3/*6 samples (ID1233). The c.55_56insGAGTCG insertion is shown by a red dotted line; M, 1 kb DNA marker; kb, kilobase; NTC, no template control.

To define the *NUDT15* diplotype of patients harboring heterozygous c.55_56GAGTCG and c.415C > T, an AS-PCR assay was used for the c.415C or c.415T alleles. The common forward primer (CP-F) was specific for the 5′ UTR and the reverse primer was specific for c.415C (415C-R) or c.415T (415T-R) (Fig. 1B). To improve the mismatch discrimination, the 3′ nucleotide of the reverse primer was substituted with LNA. Several nucleotides of the forward and reverse primers were also replaced with LNA to increase the melting temperature (Tm) to meet the parameters of PCR. Unlike PCR with LNA primers, AS-PCR using DNA primers cannot distinguish between the c.415C and c.415T alleles (Fig. 3). To test the efficacy of AS-PCR assay, samples with homozygous c.415C (*1/*1), homozygous c.415T (*2/*3), and heterozygous c.415C > T (*1/*2 and *3/*6) were used. The *1/*1 and *2/*3 samples could only be amplified using 415C AS-PCR or 415T AS-PCR, respectively, while *1/*2 and *3/*6 could be amplified using both AS-PCRs (Fig. 4). The sequencing of the AS-PCR products using the E1-S primer to determine the *NUDT15* diplotype, located the c.55_56insGAGTCG variant on the c.415T allele of the *1/*2 sample or the c.415C allele of the *3/*6 sample (Fig. 4).

### Three patients with compound heterozygous variants were identified

Of the 63 patients, 60 harbored NUDT15*1/*2, and two samples were identified to harbor NUDT15*3/*6 (Fig. 4, Fig. S1) and one NUDT15*2/*7 (Fig. S2). The two patients harboring NUDT15*3/*6 identified in this study (ID898 and ID1233) included the patient (ID898) reported in our previous study^16^. Unfortunately, the patient (ID1233) identified in this study died of fungal infection during the induction chemotherapy and took 6-MP only for 3 days. Therefore, we are not able to determine her tolerable dose of 6-MP.”

## Discussion

The emerging role of *NUDT15* in precision medicine has been indicated in several guidelines, which has rendered the evaluation of this gene important^11,22^. Most laboratories perform Sanger sequencing to screen the genetic variants of *NUDT1*5^22^. If c.55_56insGAGTCG and c.415C > T are identified simultaneously, the mutation is labeled as NUDT15*1/*2. However, NUDT15*3/*6 may also exist in some cases. Our study showed that AS-PCR targeting the C or T nucleotide amplifies the individual allele and can be used to differentiate NUDT15*1/*2 from NUDT15*3/*6. This approach might help to precisely identify the genetic variants of *NUDT15* and determine the allele type. Therefore, it could be more appropriate to determine the dosing of mercaptopurine before prescribing it for treating childhood ALL.

The NUDT15*6 allele is associated with uncertain functions and is characterized by the in-frame insertion of a 6 base pair repeat (GAGTCG) in exon 1 of *NUDT15*, which results in four copies of the GAGTCG repeat. The NUDT15*6 allele is present in approximately 1.3% of the East Asian population^23^. Detection of the duplication is required to accurately detect and distinguish NUDT15*2 from NUDT15*3^22^. Earlier, three studies investigated the diplotype of *NUDT15*. Yu et al. used targeted sequencing of *NUDT15* cDNA to determine the diplotype, and two patients harboring compound heterozygosity were identified, one with NUDT15*3/*6 and the other with NUDT15*2/*7^16^. Tsujimoto et al. defined the diplotypes of 14 patients harboring the two variants (i.e., c.415C > T and c.55_56insGAGTCG) using droplet digital PCR (ddPCR); however, they could not identify any patients with NUDT15*3/*6^24^. Recently, although the robustness of a long-read HiFi amplicon sequencing assay has been reported for phased full gene characterization and the discovery of pharmacogenomic allele^25^, the method may not conveniently elucidate the phasing of *NUDT15* in daily clinical practice due to its higher cost and unavailability in most laboratories in Taiwan.

AS-PCR has several advantages in identifying the *NUDT15* diplotype in daily clinical practice. Usually, physicians need to send one sample to the laboratory to assess the mutations in *NUDT15* before prescribing mercaptopurine or related drugs. Sanger sequencing is a rapid method for screening variants of the *NUDT15* coding region. If variants are not identified in the coding region, the genotype should be reported as wild type and exempted from further analysis. However, if c.415C > T and the c.55_56insGAGTCG are identified simultaneously, AS-PCR with LNA primers can be used to determine the diplotype, which will be cost-effective (approximately 20 USD per sample) and fast (TAT within 5 working days). Importantly, the process is lesser labor-intensive than ddPCR or NGS-based methods^16,24,25^.

The patients with NUDT15*3/*6 are believed to have intermediate-poor metabolism and might require a dose reduction of 6-MP^11^. A recent publication of *NUDT15* by several joint committees suggested NUDT15*3 as a Tier 1 variation^22^. Tier 1 alleles include the most common variant alleles that can be used to maximize disease detection rate in most populations. Variant alleles associated with the loss-of-function present in 0.01% or greater percentage of any subpopulation are included in Tier 2 for more comprehensive genotyping. NUDT15*2, *4, *6, *9 and *14 are listed as Tier 2 variations^22^. Many laboratories that perform *NUDT15* genotyping only target the c.415C > T variant present in both NUDT15*2 and *3 and may not differentiate between these two alleles, as it is technically challenging to assess c.55_56insGAGTCG. However, c.55_56insGAGTCG can be identified using Sanger sequencing. AS-PCR using LNA primers can differentiate between NUDT15*1/*2 or *3/*6. As patients harboring NUDT15*3/*6 can be identified in high variant allele frequency populations, differentiating between these two genotypes might be important if more cases were identified and their lower tolerable 6-MP doses were verified. In our previous study, we identified 3 patients with *NUDT15* compound heterozygous variant^16^. The tolerable 6-MP doses were lower for NUDT15*3/*6 (2.5 mg/m^2^, one patient died before 6-MP dose evaluation) and NUDT15*2/*7 (6.7 mg/m^2^) than that for NUDT15*1/*2 (median, 12.5 mg/m^2^)^16,19^. The patients with NUDT15*3/*6 or NUDT15*2/*7 still experienced frequent neutropenia or neutropenic fever even though they were given a low dose of mercaptopurine (12 mg/m^2^). Because white blood cell count-based dose adjustments are regularly performed for known NUDT15- deficient patients and result in a reduced risk of neutropenia and febrile neutropenia^26^, the accurate determination of patients with NUDT15*3/*6 may avoid them from receiving a high dose of mercaptopurine; even if the mercaptopurine dose was already reduced based on the results of NUDT15*1/*2 genetic analysis. The clinical significance of NUDT15*3/*6 needs further evaluation in future international clinical trials^27^ due to the lower incidence rate and challenges in detecting this variant in Asian populations.

In conclusion, Sanger sequencing can be used to screen *NUDT15* variants. In contrast, AS-PCR with LNA primers can be applied to determine the true diplotype of *NUDT15* and simultaneously detect heterozygous c.55_56insGAGTCG and c.415C > T. This approach would enable the diagnosis of compound heterozygous mutations (NUDT15*3/*6). However, because of the limited number of NUDT15*3/*6 cases, and as most methods are unable to differentiate them from NUDT15*1/*2, the tolerable dosing of 6-MP remains to be determined for this small subset of patients. In the future, large international clinical trials are required to evaluate the adjusted dosing of mercaptopurine in pediatric ALL patients with NUDT15*3/*6.

## Supplementary Information

Supplementary Information.

## Data Availability

The datasets generated during and/or analyzed during the current study are available from the corresponding author upon reasonable request.
