# Supplementary material for: Allele-specific polymerase chain reaction can determine the diplotype of NUDT15 variants in patients with childhood acute lymphoblastic Leukemia
Source: Sci Rep. 2023 Jan 10;13:490. doi: 10.1038/s41598-023-27720-2 (PMC9832159; doi:10.1038/s41598-023-27720-2)
Supplement: Supplementary file 1 — Supplementary Information. [file 41598_2023_27720_MOESM1_ESM.pdf]

Allele-specific Polymerase Chain Reaction can Determine the Diplotype of *NUDT15* Variants in Patients with Childhood Acute Lymphoblastic Leukemia

Chih-Hsiang Yu[1], Ya-Hsuan Chang[1], Der-Shiun Wang[2,3], Shiann-Tarng Jou[4,5], Chien-Yu Lin[1], Kai-Hsin Lin[4], Meng-Yao Lu[4,5], Kang-Hsi Wu[6], Chao-Neng Cheng[7], Hsiu-Hao Chang[4,5], Shu-Wei Chou[4], Min-Yu Su[8], Yu-Ling Ni[9], Pei-Yuan Xu[9], Dong-Tsamn Lin[4,9], Shu-Wha Lin[10], Hsuan-Yu Chen[1], Yung-Li Yang[4,9,11]

[1]Institute of Statistical Science Academia Sinica, Taipei, Taiwan

[2]Department of Pediatrics, Tri-service General Hospital, Taipei, Taiwan

[3]Graduate Institute of Clinical Medicine, College of Medicine, National Taiwan University, Taipei, Taiwan

[4]Department of Pediatrics, National Taiwan University Children's Hospital, Taipei, Taiwan

[5]Department of Pediatrics, College of Medicine, National Taiwan University, Taipei, Taiwan

[6] Department of Pediatrics, Chung Shan Medical University Hospital and School of Medicine, Chung Shan Medical University, Taichung, Taiwan

[7]Department of Pediatrics, National Cheng Kung University Hospital and Department

of Pediatrics, College of Medicine, National Cheng Kung University, Tainan, Taiwan, Tainan, Taiwan

[8]Department of Pediatrics, China Medical University Children's Hospital, Taichung, Taiwan

[9]Department of Laboratory Medicine, National Taiwan University Hospital, Taipei, Taiwan

[10]Departments of Clinical Laboratory Sciences and Medical Biotechnology, National Taiwan University, Taipei, Taiwan

[11]Department of Laboratory Medicine, College of Medicine, National Taiwan University, Taipei, Taiwan

Corresponding author:

Yung-Li Yang

Department of Laboratory Medicine, National Taiwan University Hospital,

No. 7, Chung Shan S. Rd, Zhongshan S. Rd, Taipei, 100, Taiwan, R.O.C.

Tel.: +886-2-23123456-71712

Fax: +886-2-23224263

E-mail: yangyl92@ntu.edu.tw

Keywords: Adverse drug reactions, Pharmacogenomics, Pediatric ALL, NUDT15

Running title: *NUDT15* diplotyping by AS-PCR

Funding

This work was supported by grants from the Ministry of Science and Technology, Taiwan (110-2314-B-002-091-MY3 to YLY), and the National Taiwan University Hospital (110-L1007, to YLY).

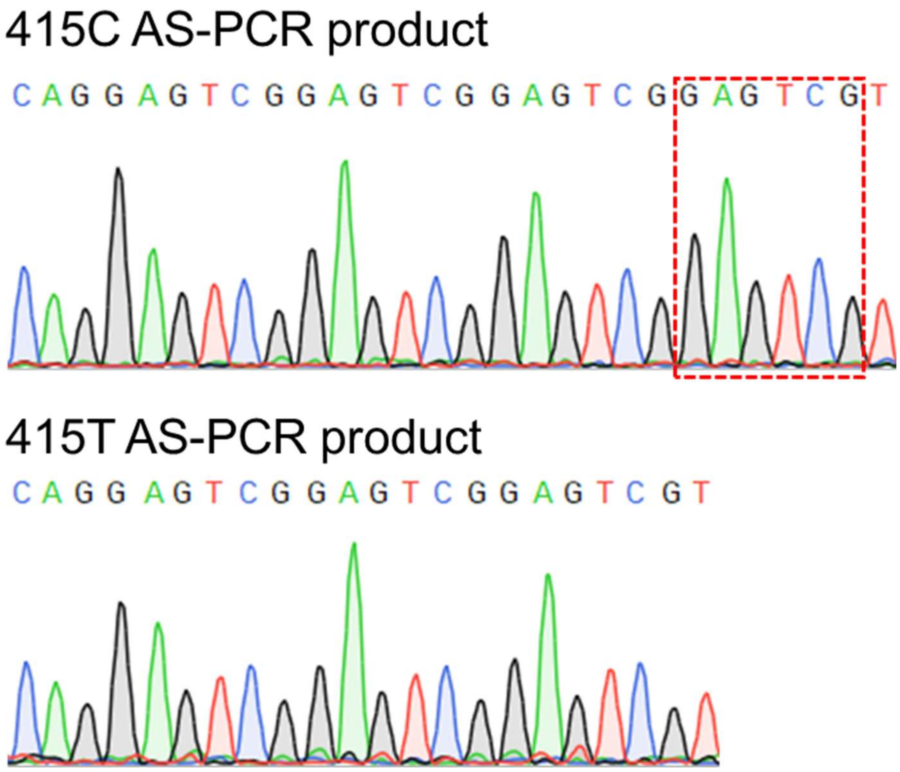

Supplementary Figure 1. Results of Sanger sequencing of AS-PCR product of *NUDT15**3/*6 (ID898). c.55_56insGAGTCG is located on c.415 allele (*NUDT15**6). c.55_56insGAGTCG insertion is shown by a red dotted line.

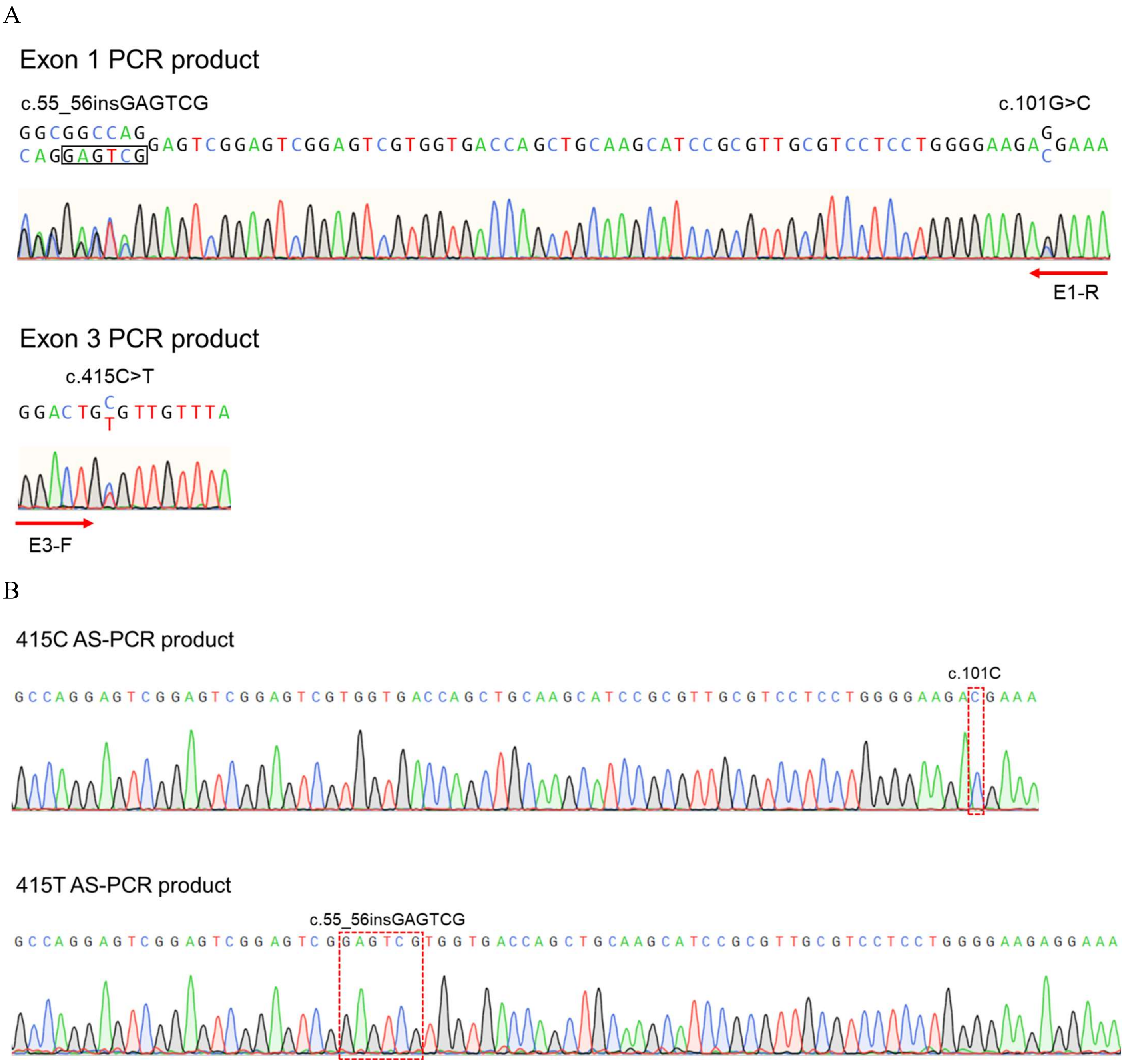

Supplementary Figure 2. The sequencing results of *NUDT15**2/*7 (ID341). (A) The *NUDT15* exon 1 and exon 3 was amplified and sequenced using E1-R or E3-F primer respectively. The sequencing direction was represented by red arrow. Three heterozygous *NUDT15* variants, c.55_56insGAGTCG, c.101G>C and c.415C>T were identified. (B) *NUDT15* diplotype analysis using allele-specific PCR. The c.101C variant was located on c.415C allele (*NUDT15**7) and c.55_56insGAGTCG variant was located on c.415T allele (*NUDT15**2). The variants are shown by a red dotted line.

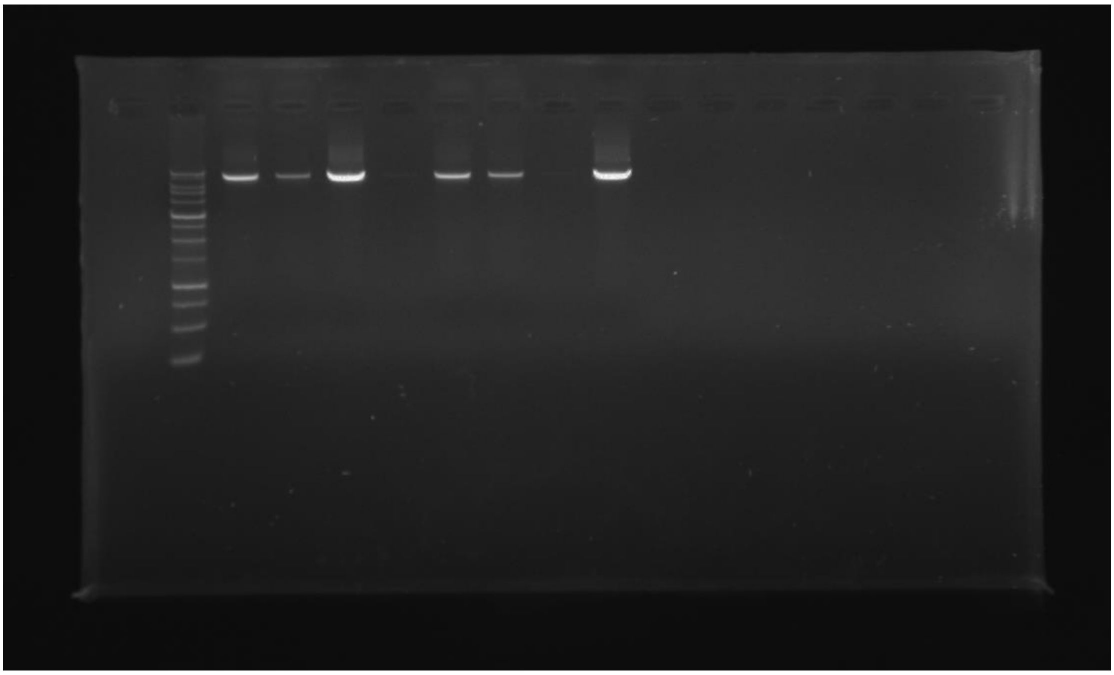

Supplementary Figure 3. Agarose gel image which are shown cropped in Figure 3

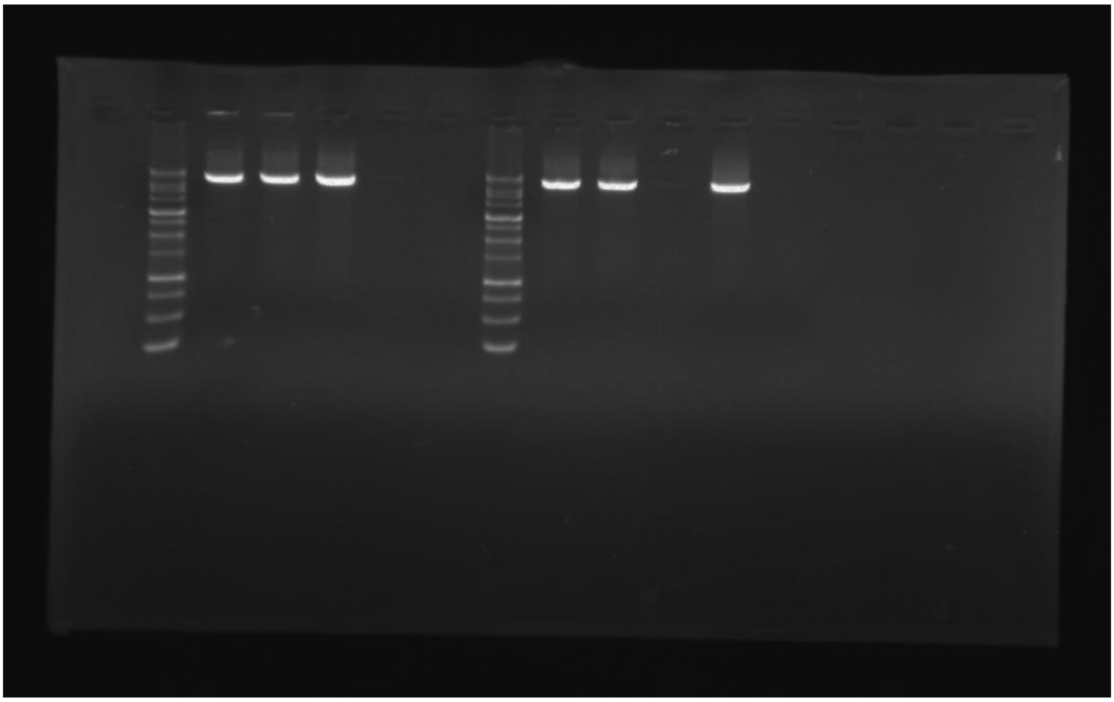

Supplementary Figure 4. Agarose gel image which are shown cropped in Figure 4A
